# 

**Published:** 1874-04

**Authors:** 


### CONTENTS.

### COMMUNICATIONS.

A Biographical Sketch of Dr. John Allen.............. 141
Sensitive Dentine and its Remedy.................... 148

### PROCEEDINGS OF SOCIETIES.

Discussions....................................... 150

Eastern Ohio Dental Association.................... 162

### SELECTIONS.

Law and Medicine.................................. 166
Mortality of Various Cities........................ 167
A Bad Case........................................ 167
A Hint on Giving Iodide of Potassium............... 168
Nickel Plating..................................... 169
The Magic Lantern as an aid to Instruction in Chemical and Physical Lectures............ 172
Production of Iodine in France..................... 174
Good Potable Water................................. 175
Selected Abstracts................................. 176
Oriental Wisdom.................................... 177
A Test for Carbolic Acid........................... 178

### MISCELLANEOUS.

A Letter........................................... 178

Mad River Valley Dental Society.................... 179

### EDITORIAL.

The Health of the Dentist.......................... 180
Meeting of the Mississippi Valley Dental Society....... 183
Bibliographical.................................... 184
Obituary........................................... 184

